# The Effect of *Enterococcus faecium* AL41 on the Acute Phase Proteins and Selected Mucosal Immune Molecules in Broiler Chickens

**DOI:** 10.3390/life12040598

**Published:** 2022-04-18

**Authors:** Viera Karaffová, Csilla Tóthová, Renáta Szabóová, Viera Revajová, Andrea Lauková, Zuzana Ševčíková, Róbert Herich, Rudolf Žitňan, Martin Levkut, Mikuláš Levkut, Zita Faixová, Oskar Nagy

**Affiliations:** 1 Department of Morphological Disciplines, University of Veterinary Medicine and Pharmacy in Košice, Komenského 73, 041 81 Košice, Slovakia; viera.revajova@uvlf.sk (V.R.); zuzana.sevcikova@uvlf.sk (Z.Š.); robert.herich@uvlf.sk (R.H.); martin.levkut@uvlf.sk (M.L.); mikulas.levkut@uvlf.sk (M.L.); 2 Clinic of Ruminants, University of Veterinary Medicine and Pharmacy in Košice, Komenského 73, 041 81 Košice, Slovakia; csilla.tothova@uvlf.sk (C.T.); oskar.nagy@uvlf.sk (O.N.); 3 Department of Biology and Physiology, University of Veterinary Medicine and Pharmacy in Košice, Komenského 73, 041 81 Košice, Slovakia; renata.szaboova@uvlf.sk (R.S.); zita.faixova@uvlf.sk (Z.F.); 4 Centre of Biosciences of the Slovak Academy of Sciences, Institute of Animal Physiology, Šoltésovej 4-6, 040 01 Košice, Slovakia; laukova@saske.sk; 5 National Agriculture and Food Centre, Research Institute for Animal Production Nitra, Hlohovecká 2, 951 41 Lužianky, Slovakia; rudolf.zitnan@nppc.sk; 6 Institute of Neuroimmunology, Dúbravská Cesta 9, 845 10 Bratislava, Slovakia

**Keywords:** *Enterococcus faecium* AL41, acute phase protein, probiotic bacteria, mucosal immune response, broiler chickens

## Abstract

Probiotic bacteria, including the *Enterococcus faecium* strain, can improve intestinal mucosal health by several mechanisms, including modulation of the immune response, as well as by improving the protective function of the epithelial barrier. In this study, we tested the effect of *Enterococcus faecium* AL41 on the acute phase proteins response (blood), gene expression of selected molecules of mucosal immunity (immunoglobulin A, mucin-2, insulin-like growth factor 2) and mucus production (all parts of the small intestine) in broilers. Eighty broiler chicks were divided into two groups: a control and *E. faecium* AL41 (birds were inoculated with AL41 for 7 days) group. The whole experiment lasted 11 days. Our results revealed that the administration of *E. faecium* AL41 had no substantial effect on the concentrations of acute phase proteins, but we recorded a significant increase in β- and γ-globulin fractions at the end of the experiment, which may indicate an improvement in the immune status. A significant prolonged stimulatory effect of *E. faecium* AL41 on the relative expression of molecules (immunoglobulin A, mucin-2) as well as on the dynamic of mucus production in the chicken intestine was observed. In addition, AL41 significantly reduced the total number of enterococci in the cecum and faeces.

## 1. Introduction

The mucosal immune system represents a separate part of the immune system that provides local immunity in the mucous membranes of the gastrointestinal as well as respiratory tracts. It essentially tolerates commensal microbes and at the same time responds quickly and effectively to pathogenic organisms [1]. The major gel-forming mucin (MUC-2) forms the primary barrier component of the mucus layers and represents the main site for secretory immunoglobulin A (IgA). The polymeric Ig receptor, which is expressed on the basolateral surface of epithelium, is used to transport polymeric IgA from the lamina propria to the luminal mucins to form the first lines of intestinal defence. IgA together with MUC-2 limit epithelial contact with pathogens and other potentially dangerous antigens and their penetration [2]. On the other hand, it selectively facilitates the adherent growth of normal intestinal microbiota [3]. Insulin-like growth factor 2 (IGF2) together with IGF-1 are known as intestinotropic factors mainly for the small intestinal epithelium [4].

Probiotics can improve intestinal mucosal health through several mechanisms, including the production of antimicrobials, short-chain fatty acids, modulation of the immune response, as well as competitive elimination of pathogenic bacteria, thereby enhancing epithelial barrier function [5]. Intestinal mucus layer is the first line of defence protecting epithelium against luminal threats including mechanical forces during the digestion process, enzymes and gut bacteria. The intestinal mucus also plays important roles in supporting the colonization with commensal bacteria, maintaining an appropriate environment for digestion and facilitating nutrient transport from the lumen to the underlying epithelium [6]. Intestinal morphological measurements, such as increased villus height, short crypt depth and higher villus height–crypt depth ratio indicate an increase in nutrient absorption by increasing the available surface area for nutrient absorption. The proliferation and differentiation of goblet cells affect the mucosal integrity and dynamic to maintain mucus thickness [6]. The amount of mucus production depends on the number of goblet cells in the intestinal villi and crypts, which is a health indicator of the intestine as these cells produce mucin and exclude harmful pathogens from adhesion to the intestinal epithelium [7].

Different probiotic strains’ (*Lactobacillus casei*, L. acidophilus, *Bifidobacterium thermophilum*, *Bacillus subtilis*, *Enterococcus faecium*) influence on the gut’s histomorphology changes has been studied. This result suggested that the addition of the mentioned probiotic strains can enhance the intestinal nutrient absorption and mucus production as well as intestinal architecture [8,9].

In addition, the demand for alternative feed additives for broilers such as probiotics, prebiotics, enzymes, organic acids, herbs and their extracts has increased in recent years due to their impact on productivity and animal health [10].

Enterococci are among the lactic acid bacteria (LABs), which include pathogenic and commensal microorganisms ubiquitous in the environment, even as intestinal symbionts of animals and humans. In addition, several *Enterococcus* strains are reported to produce antimicrobial compounds, including bacteriocins. Bacteriocin production is currently considered a probiotic property [11]. Currently, the enterococcal strains *E. faecium* and *E. faecalis* are the only enterococci used as probiotics or feed additives [12].

*E. faecium* AL41 is an enterocin M bacteriocin-producing probiotic strain belonging to the Firmicutes phylum, which fulfils EFSA rules [13,14].

The dietary supplementation with enterococcal probiotics may improve health and growth performances through the optimal utilization of nutrients and maintenance of intestinal integrity, and may reduce the death rate by regulating immune responses in broiler chickens [15]. One of the ways to monitor the health state, as well as assess metabolic alterations related to protein profile and immune responses, is the evaluation of acute phase reactants. Acute phase reactants are a group of proteins whose serum concentrations change in response to any injury, disturbances in homeostasis or stress as part of the non-specific innate immune response [16]. In addition to the determination of these specific proteins, serum protein electrophoresis could be of great diagnostic importance to describe the distribution of serum proteins and to assess the changes, especially in the gamma-globulin fraction caused by the overproduction of a single or a group of immunoglobulins [17]. Even though acute phase proteins may be relevant biomarkers of the health state, there are very few studies assessing the effect of probiotic supplementation on their synthesis.

In our previous experiments with broiler chicks, the administration of *E. faecium* AL41 strain resulted mainly in an immunomodulatory effect on cytokine expression during *Salmonella* and *Campylobacter* infections and increased the concentration of secretory IgA in the intestine flush [18,19]. Therefore, we decided to observe the effect of strain AL41 on the acute phase proteins response, distribution of blood serum proteins and important parameters of mucosal gut immunity in broiler chicks.

## 2. Materials and Methods

### 2.1. Experimental Scheme

#### Animals

The chickens were handled and killed in accordance with state regulations. The specific experiment was approved by the Ethics Committee of the Veterinary Medicine and Pharmacy in Košice followed by the Committee for Animal Welfare of the Ministry of Agriculture of the Slovak Republic (permit number 1184-3/2020-220).

Eighty 1-day-old COBB 500 male cock chicks were divided into two groups (*n* = 40). The following experimental groups were included in the study: the control group and the EF group, where birds were inoculated with *E. faecium* AL41 (CCM 8558). The experiment lasted 11 days. The chickens were placed in 4 hardwood pens with an area of 2.06 m^2^ (length 165 cm, width 125 cm, height 120 cm) covered with wood and fed a standard BR-1 compound feed (BR1, Čaňa, Košice, Slovakia) (Table 1) with access to water ad libitum. The broilers were kept at an ambient temperature of 30–32 °C with a relative humidity of 40–80% throughout the experiment, in a light/dark mode for 12 h. The control of the temperature and humidity in the room was performed 8 times a day (every 3 h) with a KlimaLogg Pro monitoring device with a signalling system. Environmental conditions were kept following the broiler breeding criteria [20]. Prior to the start of the experiment, faecal control samples were taken from the chickens for microbiological examination. The blood samples (zero day and 11th day of the experiment) and the samples from all individual parts of the small intestine (5th, 8th and 11th day of the experiment) were collected from 10 chickens from both groups in each sampling.

### 2.2. Preparing of Probiotic Strain

The probiotic strain of *E. faecium* AL41 was grown as previously detailed by Karaffová et al. [21]. A suspension of *E. faecium* in dose 10^9^ CFU/0.2 mL was supplemented individually perorally to the chickens in the EF group daily, from the first to the seventh day of the experiment. To simulate the same manipulation stress, an equal volume of saline was applied to the control group with a Pasteur pipette.

### 2.3. Laboratory Analyses

The blood samples from chickens (on day 0 and at the end of the experiment—11th day) were taken into 1.1 mL serum gel separator tubes without additives and anticoagulants (Sarstedt, Nümbrecht, Germany). After letting the blood samples coagulate at room temperature, sera were separated by centrifugation at 3000× *g* for 15 min and then transferred into Eppendorf tubes. The serum samples were immediately processed and analysed, and aliquots were kept frozen at −20 °C for further laboratory analyses. The serum samples were analysed for the concentrations of total serum proteins (TP, g/L), the electrophoretic pattern of serum proteins and selected acute phase proteins. The biuret method was applied to measure the TP concentrations using commercially available diagnostic kits (Randox, Crumlin, UK) and the automated chemistry analyser Alizé (Lisabio, Pouilly en Auxois, France). The separation and distribution of serum protein fractions were performed by zone electrophoresis on agarose gel using an automated electrophoresis system Hydrasys with commercial diagnostic kits Hydragel 7 Proteine (Sebia Corporate, Lisses, Evry Cedex, France) [22].

The protein fractions were expressed as relative values (%) according to the optical density and their absolute concentrations (g/L) were quantified from the TP concentrations. Albumin–globulin ratios (A/G) were calculated as well. The concentrations of serum amyloid A (SAA, ng/mL) were quantified by double antibody sandwich enzyme-linked immunosorbent assay (ELISA) using a commercially available Chicken SAA ELISA kit (Immunology Consultants Laboratory, Inc., Portland, OR, USA). Haptoglobin (Hp, mg/mL) was measured spectrophotometrically using commercial colorimetric kits (Tridelta Development, Kildare, Ireland) in microplates.

#### 2.3.1. Homogenization of Jejunal Samples and Isolation of Total RNA of IgA, MUC-2 and IGF-2 (Growth Factor) Gene

Samples of jejunum (20 mg weighted pieces) were immediately placed in RNA later solution (Qiagen, UK) and stored at −70 °C before RNA purification and reverse transcription as mentioned in Karaffová et al. [23].

#### 2.3.2. Relative Expression of Genes in Quantitative Real-Time PCR (qRT-PCR)

The mRNA levels of IgA, MUC-2 and IGF-2 genes were determined. Additionally, mRNA relative expression of the reference gene, coding GAPDH (glyceraldehyde-3-phosphate dehydrogenase), was selected based on confirmed expression stability using the geNorm program. The primer sequences, annealing temperatures and times for each primer used for qRT-PCR are listed in Table 2. All primer sets allowed cDNA amplification efficiencies between 94% and 100%.

Amplification and detection of target products were performed using the CFX 96 RT system (Bio-Rad, Hercules, CA, USA) and Maxima SYBR Green qPCR Master Mix (Thermo Scientific, Waltham, MA, USA). Subsequent qRT-PCR to detect relative expression of mRNA of selected genes was performed for 36 cycles under the following conditions: initial denaturation at 95 °C for 2 min, subsequent denaturation at 95 °C for 15 s, annealing (Table 2) and final extension step for 2 min at 72 °C. A melting curve from 50 °C to 95 °C with readings at every 0.5 °C was generated for each individual qRT-PCR plate. All reactions were conducted in triplicate. We also confirmed that the efficiency of amplification for each selected gene was essentially 100% in the exponential phase of the reaction, where the quantification cycle (Cq) was calculated. The Cq values of the studied genes were normalised to an average Cq value of the reference gene (ΔCq), and the relative expression of each gene was calculated mathematically as 2^–ΔCq^.

### 2.4. Mucus Production

The duodenum, jejunum and ileum samples were obtained from randomly selected animals (*n* = 6). After exsanguinations from the small intestine, different segment samples were processed in duplicates for mucus determination according to Smirnov et al. [25] and modified by Faixová et al. [28]. The amount of produced mucus was determined by the ELISA assay technique (Apollo LB 913, Berthold Technologies, Bad Wildbad, Germany) at the wavelength 630 nm using the software PhotoRead version 2.2.2.1 (Apollo LB 913, Berthold Technologies, Bad Wildbad, Germany). The mucus production quantity results were expressed in grams (g ± standard deviation-SD).

### 2.5. Microbiology

In the experiment, the so-called rifampicin-labelled strain AL41 = CCM 8558 [13] was used to distinguish it from other enterococcal microbiota in the faeces (on 0, 8th and 11th day of the experiment) and in the cecum (8th and 11th day of the experiment). M-Enterococcus agar (M-Enterococcus agar, Difco) supplemented with rifampicin (100 μg) was used to capture the number of strain AL41 = CCM 8558. Total enterococcal counts were determined using M-Enterococcus agar (Difco, Sparks, MD, USA) and coliform bacteria were isolated on MacConkey agar (Difco, Sparks, MD, USA). All plates were cultured according to the genera at 37 °C for 24 h (in a partially anaerobic atmosphere). To eliminate the presence of *Campylobacter* and *Salmonella*, Rappaport-Vassiliadis Broth (Merck) was used to capture *Salmonella* sp., followed by seeding on Brilliant green agar (Becton and Dickinson, Cockeysville, MD, USA). CM0935 Campylobacter agar base (Karmali) supplemented with Campylobacter Selective Supplement (Karmali) SR0167 (Oxoid Ltd., Basingstoke, UK) was used to exclude the presence of *Campylobacter* sp. when cultured in an anaerobic box and at a temperature of 42 ℃. The faeces samples were processed by a standard microbiological method (ISO) and were grown on the media mentioned above. The bacterial counts were expressed in colony-forming units as CFU/g^−1^ ± sd.

All samples were free of *Salmonella* sp. and *Campylobacter* sp.

### 2.6. Statistical Analyses

All statistical analyses were carried out by the statistical software GraphPad Prism 8.3 (GraphPad Software Inc., San Diego, CA, USA). Kolmogorov–Smirnov test for normality was used to evaluate the distribution of the data. The statistical model of the unpaired T-test was applied to compare the means related to the two sample collections and to determine the significance of differences between the sample collection days in both groups. For other parameters, differences between the control and experimental groups were tested also by the unpaired *t*-test. The levels of statistical significance were expressed as *p*-value (*p* < 0.05, *p* < 0.01, *p* < 0.001). Values in figures are given as means resp. medians in the case of relative gene expression with standard deviations (±SD).

## 3. Results

### 3.1. Laboratory Analyses

As presented in Table 3, the analyses of the concentrations of Hp showed no significant differences between the days 0 and 11 in the control or in the experimental group of chickens. The concentrations of SAA were slightly but non-significantly higher on day 11 of the experiment in the control, as well as in the experimental chickens. The concentrations of total serum proteins were significantly higher on day 11 of the experiment when compared to day 0 in the control, as well as in the experimental group of chickens (*p* < 0.01 and *p* < 0.05, respectively). Serum protein electrophoresis identified six protein fractions in broiler chickens, including prealbumin, albumin, α_1_-, α_2_-, β- and γ-globulins. On day 11 of the evaluation, the relative concentrations of protein fractions showed a slightly non-significantly higher proportion of prealbumin in the experimental group compared to the control group. Albumin was the most prominent protein fraction and formed nearly 44% of total serum proteins in both groups of chickens. On day 11 of the evaluated period, its values were slightly lower in the experimental chickens than in the chickens of the control group. No significant differences in the relative concentrations of α_1_-globulins were found between the sample collections in the control or the experimental group. In the relative concentrations of α_2_-globulins, significant differences between the sample collections were obtained. Their values were significantly lower on day 11 of the experiment in both groups of animals as compared to day 0 (*p* < 0.05). While the mean relative concentrations of β-globulins in the control group were similar on days 0 and 11 of the experiment, their proportion in the experimental group was non-significantly higher on day 11. The relative values of γ-globulins were higher on day 11 of the experiment in both groups of chickens, and this difference was significant in the experimental group (*p* < 0.01). The A/G ratios on day 11 were non-significantly lower on day 11 than on day 0 in both groups of chickens.

The absolute concentrations of prealbumin in the control group were approximately similar on days 0 and 11 of the evaluation; in the experimental group, the values obtained on day 11 were non-significantly higher. A trend of higher values on day 11 was also observed in the absolute concentrations of albumin; the differences in mean values between the sample collections were significant in the control group of chickens (*p* < 0.01). No significant differences were found in the absolute concentrations of α_1_- and α_2_-globulins between the sample collections of both groups of chickens. Differences between the sample collections occurred in the absolute concentrations of β-globulins, where the values obtained on day 11 in the experimental group of chickens were significantly higher than on day 0 (*p* < 0.01). The absolute concentrations of γ-globulins found in both groups of chickens on day 11 were significantly higher when compared to those on day 0. However, on day 11, a significantly higher mean γ-globulin value was recorded in the experimental group of chickens (*p* < 0.01).

### 3.2. Relative Expression of Genes in qRT-PCR

**Figure 1:**
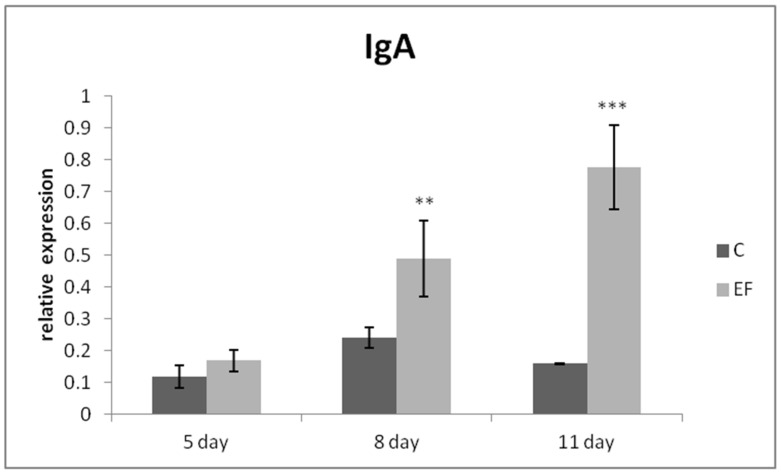
Relative expression of IgA gene in the jejunum of chickens treated with *E. faecium* AL41. Results at each time point are the median of 2^–ΔCq^. Means with different superscripts are significantly different ** *p* < 0.01; *** *p* < 0.001.

**Figure 2:**
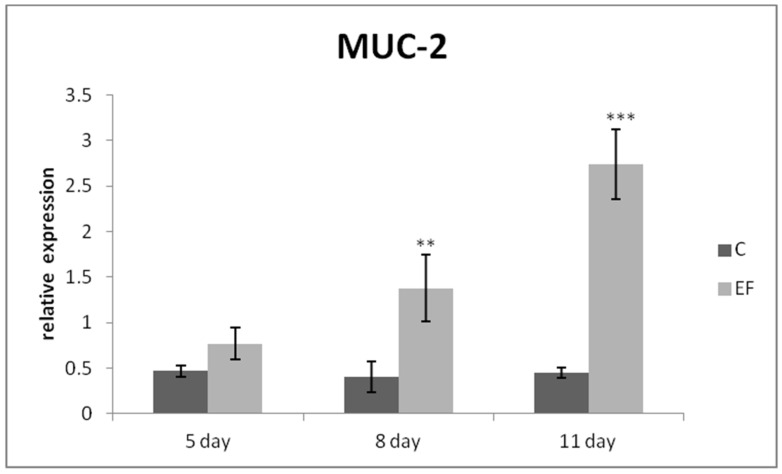
Relative expression of MUC-2 gene in the jejunum of chickens treated with *E. faecium* AL41. Results at each time point are the median of 2^–ΔCq^. Means with different superscripts are significantly different ** *p* < 0.01; *** *p* < 0.001.

**Figure 3:**
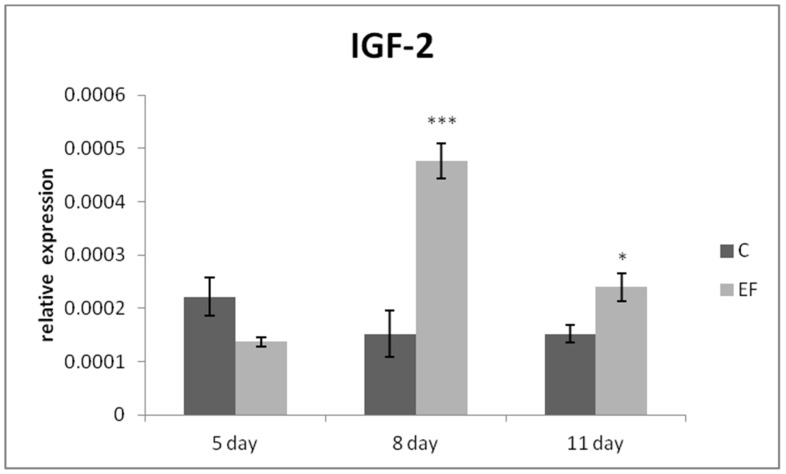
Relative expression of IGF-2 gene in the jejunum of chickens treated with *E. faecium* AL41. Results at each time point are the median of 2^–ΔCq^. Means with different superscripts are significantly different * *p* < 0.05; *** *p* < 0.001.

The relative expression of the IgA gene was markedly upregulated mainly on day 8 (*p* < 0.01), as well as day 11 in the EF group as compared to the control (*p* < 0.001) (Figure 1). The same tendency was recorded for MUC-2 gene expression, which was higher in the EF group on days 8 and 11 than in the control (*p* < 0.01; *p* < 0.001) (Figure 2). The relative expression of growth factor IGF-2 was upregulated in the experimental group in comparison with the control in the last two sampling days (*p* < 0.05; *p* < 0.001) (Figure 3).

### 3.3. Mucus Production

Mucus production was significantly increased in the duodenum (*p* < 0.05) as well as in the jejunum (*p* < 0.01) at the end of the experiment (day 11) in comparison with mucus production in the duodenum and ileum on day 8 of the experiment. The prolonged beneficial influence on mucus production quantity was observed at the end of the experiment in the duodenum (*p* < 0.001), jejunum (*p* < 0.001) and ileum (*p* < 0.001) in the experimental group compared to the relevant control group on day 11 of the experiment (Table 4).

### 3.4. Microbial Screening

Strain AL41 = CCM 8558 alone colonized the digestive tract of chickens; when in the faeces it reached 3.0 log_10_ cfu/g in the experimental group (day 8), and on day 11 its numbers decreased only slightly. Enterococcal counts decreased significantly in the experimental group on day 8, as well as on day 11 of the experiment compared to the control (*p* < 0.05). We assume that the decrease in total enterococci in the experimental group was due to the action of *E. faecium* AL41 alone, through the production of bacteriocins, or by the production of lactic acid. Coliform counts in the faeces were not affected by the administration of *E. faecium* AL41 (Table 5).

The numbers of strain *E. faecium* AL41 on day 8 were almost the same in the cecum and in the faeces. At the end of the experiment they decreased only slightly and did not differ between cecum and faeces (day 11 of the experiment). The same as in the faeces, the numbers of other enterococci markedly decreased in the experimental group compared to the control (*p* < 0.05) on day 8. The coliform counts in the cecum were also not affected by *E. faecium* AL41 (Table 6).

## 4. Discussion

The administration of probiotics in the feed of broiler chickens has been found to have a positive effect on the organism, manifested by improved health state and growth performances due to immunostimulation, the competitive exclusion of gut pathogens and a positive impact on the diversity and stability of intestinal microbiota [29,30]. However, the published data evaluating the effect of probiotics on the acute phase response are limited. The most important acute phase proteins in chickens are α_1_-acid glycoprotein, serum amyloid A, ceruloplasmin, transferrin, haptoglobin, fibrinogen and fibronectin [31]. The study conducted by Kefal and Toker [32] suggested that two commercial probiotic preparations—Broilact (enterococci, lactobacilli) and Bioplus 2b (*Bacillus licheniformis, Bacillus subtilis)* alone do not affect the serum concentrations of ceruloplasmin, transferrin and fibrinogen in broilers exposed to *Salmonella typhimurium* lipopolysaccharides. Similarly, the results obtained in our study showed no significant influence of the dietary administration of *E. faecium* AL41 on the concentrations of inflammatory markers in chickens, as no significant differences in the concentrations of haptoglobin and serum amyloid A were found between days 0 and 11 of the experiment. A significant increase in total protein values, as well as markedly higher concentrations of albumin on day 11, was recorded in both groups of animals. This might be associated with normal growth processes and feeding with protein-rich diets during the fattening period [33]. Furthermore, the increase in total serum proteins might be related to the redistribution of nutrients away from the immune response and acute phase protein synthesis, which resulted in the increased availability of nutrients for growth and development [34]. The increase in total serum proteins was accompanied by significantly higher proportions of β- and γ-globulin fractions mainly in the experimental chickens (EF group). A significant increase in γ-globulins was observed in control animals as well, but the increase was less evident in chickens fed without fodder recipes containing probiotics. Cetin et al. [35] reported that the supplementation of feed with probiotics resulted in elevated concentrations of immunoglobulin G and M in turkeys, which have been linked to better growth performance and disease resistance in the evaluated animals. Immunoglobulins are the main constituent of the γ-globulin fraction, but some immunoglobulin classes (IgM and IgG) may migrate into the β-globulin region [36]. Therefore, the more significant increase in β- and γ-globulin fractions in the experimental chickens could be a result of the increased synthesis of immunoglobulins due to the feed supplementation. Similarly, Stef et al. [37] presented higher serum gamma-globulin concentrations in broiler chickens supplemented with probiotics (*Lactobacillus paracasei* J.R., *Lactobacillus rhamnosus* 15b, *Lactobacillus lactis* y, *Lactobacillus lactis* FO) and amino acids, resulting in better immune statuses and growth performances. Likewise, Dev et al. [38] concluded that the administration of *Lactobacillus acidophilus* with mannan oligosaccharides led to higher serum globulin concentrations. However, they did not evaluate the distribution of globulin fractions. In our study, the administration of probiotics had no significant influence either on the absolute concentrations of α-globulins or on the albumin fraction. Although non-significant, the concentrations of prealbumin increased more markedly in the experimental group of chickens. As prealbumin is an important nutritional marker, its increase in experimental chickens may be related to adequate protein–calorie consumption and weight gain [39].

In the lumen of the gastrointestinal tract, digestion and absorption occur with the assistance of a broad spectrum of microbial species. The absorption takes place at the brush border, which involves epithelial surface extensions. This epithelial surface contains goblet cells, which secrete mucous fluids that cover the epithelial surface and protect it from harmful intraluminal components including pathogens. The bacterial population of the intestine influences the proliferation of mucosal cells. Mucins are major components in the cytoplasmic secretory granules of goblet cells. In addition, intestinal mucus also plays an important role in supporting the colonization by commensal bacteria, maintaining an appropriate environment for digestion and facilitating nutrient transport from the lumen to the underlying epithelium. [40]. Based on our results, we can state that a significant effect (*p* < 0.001) of *E. faecium* AL41 (CCM 8558) on the dynamics of mucus production in all parts of the small intestine (duodenum, jejunum, ileum) was recorded at the end of the experiment.

Hence, it is clear that probiotic supplementation in poultry production alters the microenvironment of the intestine and can induce alterations in mucin dynamics in the gastrointestinal tract of chickens [41]. In agreement with the previous statement, our results revealed the most significant upregulation of gene expression for MUC-2 and IgA on the last sampling (day 11), which may confirm the cumulative effect of the continuous administration of *E. faecium* AL41. Moreover, we observed the same trend in the quantification of mucin production in individual sections of the chickens’ small intestines, which was the highest on day 11 of the experiment in the EF group. These findings are very useful and relevant because intestinal bacterial homeostasis in chickens can be affected by mucin types, O-glycan composition (extent of mucin glycosylation and oligomerization) and mucus layer characteristics (inner and outer mucus thickness) [42]. In addition, MUC-2 is the predominant glycoprotein found in the small and large intestine mucus [43].

Similarly, in our previous study by Levkut et al. [44], we demonstrated that gene expression, as well as the concentration of MUC-2 and IgA in the intestinal flush from the jejunum, was markedly increased in the experimental broiler chicken group after 8 days of peroral application of synbioticum Lacto-Immuno-Vital (the product contains probiotic strains of *Enterococcus faecium* CECT 4515 and *Bacillus amyloliquefaciens* CECT 5940) compared to the control. Moreover, Aliakbarpour et al. [45] observed a significant increase in MUC-2 gene expression in broilers fed a diet supplemented with *Bacillus subtilis.*

Furthermore, we recorded the highest level of IGF-2 gene expression in the jejunum of the EF group, which is known to bind to intestinal epithelial cells and plays an important role in intestinal development [46]. In a recent study, Wu et al. [47] confirmed that supplementation with *Enterococcus faecium* NCIMB11181 in broiler feed had a notable effect on the IGF-2 gene expression as well as on other intestinal growth factors in the jejunum. These findings indicated that the addition of *E. faecium* AL41 to poultry feed strengthened the barrier function of the intestinal mucosa, as well as the parameters of the intestinal immune system.

The *E. faecium* strain AL41 sufficiently colonized the cecum of chickens in the experimental group, and at the same time reduced the numbers of total enterococci in both the cecum and the faeces in comparison to the control. The results in a study by Lauková et al. [13] showed that *E. faecium* AL41 (10^9^ CFU ml^−1^) colonized the intestine of farm ostriches in an approximately similar number and was able to control their intestinal microbiota composition. In our case, it also reduced the total enterococcal counts in the intestines as well as in the faeces. We assume that the reduction in enterococci in the experimental group was caused by the activity of the produced enterocin M (bacteriocin), which has a proteinaceous character with inhibitory activity against other enterococci [14]. The enterococcal bacteriocins are now attractive to scientists as potential drug candidates for antibiotic replacement in the treatment of multidrug-resistant pathogens. In addition, based on the observed immunomodulatory effects of *E. faecium* AL41 in this experiment, we hypothesize that the preferentially undesirable strains of enterococci were reduced.

## 5. Conclusions

The results of the presented study suggest that the administration of *E. faecium* AL41 had only negligible effect on the concentrations of evaluated acute phase proteins. On the other hand, we observed a significant increase in β- and γ-globulin fractions on day 11 of the experiment, which might indicate an improvement in the immune status. Moreover, our results revealed a significant prolonged stimulatory effect of *E. faecium* AL41 on the relative expression of all selected molecules as well as on the dynamic of mucus production in the chicken intestine. In addition, our strain AL41 significantly reduced the total numbers of enterococci in the cecum and faeces of broiler chickens. We propose that *E. faecium* AL41 is a suitable candidate for preventive administration to feed in terms of improving the intestinal mucosal barrier, which may ultimately increase the chickens’ defence against particularly intestinal pathogens.

## Figures and Tables

**Table 1:** Composition of BR1 commercial diet.

Ingredients g/kg	BR1
Wheat	290
Maize	300
Soybean meal	320
Rapeseed oil	40
Fish meal	20
Limestone	12
Dicalcium phosphate	10
Sodium chloride	2
DL-methionine	1
Vitamin-mineral mix *	5
Composition by analysis (g/kg)	
dry matter	899.9
crude protein	232.7
fat	64.5
dietary fibre	22.7
ash	53
Ca (calcium)	90.4
P (phosphorus) total	69.6

Vitamin and mineral premix *: vitamin A 12,500 IU/kg, vitamin D3 4000 IU/kg, vitamin E 80.00 mg/kg, Cu 15.00 mg/kg, vitamin D/25 cholecalciferol 1000 IU/kg, Jod 1.00 mg/kg, Mn 50.00 mg/kg, Zn 90.00 mg/kg, Fe 40.00 mg/kg, Se 30.00 mg/kg.

**Table 2:** List of primers used for the chicken gene mRNA quantification.

Primer	Sequence 5′–3′	Annealing/Temperature Time	References
IgA Fw	GTCACCGTCACCTGGACTACA	59 °C for 30 s	[24]
IgA Rev	ACCGATGGTCTCCTTCACATC		
MUC-2 Fw	GCTGATTGTCACTCACGCCTT	54 °C for 1 min	[25]
MUC-2 Rev	ATCTGCCTGAATCACAGGTGC		
IGF-2 Fw	CTCTGCTGGAAACCTACTGT	55 °C/30 s	[26]
IGF-2 Rev	GAGTACTTGGCATGAGATGG		
GAPDH Fw	CCTGCATCTGCCCATTT	59 °C/30 s	[27]
GAPDH Rev	GGCACGCCATCACTATC		

**Table 3:** Differences in the concentrations of evaluated acute phase proteins, total proteins (TP), serum protein fractions and albumin–globulin ratio (A/G) in control and experimental broiler chickens between the sample collections (mean ± SD).

Parameter		Groups of Animals			
		C (Control)		EF	
		Day 0	Day 11	Day 0	Day 11
Hp (mg/mL)		0.052 ± 0.071	0.025 ± 0.033	0.014 ± 0.013	0.022 ± 0.029
SAA (ng/mL)		34.30 ± 11.38	48.62 ± 17.16	35.41 ± 12.30	44.22 ± 6.81
TP (g/L)		26.7 ± 2.06	29.7 ± 1.46 ^b^	26.8 ± 2.19	29.1 ± 1.78 ^a^
prealb	%	1.46 ± 0.30	1.36 ± 0.30	1.53 ± 0.29	1.77 ± 0.49
	g/L	0.39 ±0.09	0.40 ± 0.08	0.41 ± 0.11	0.51 ± 0.13
alb	%	43.9 ± 1.63	43.2 ± 2.49	43.7 ± 1.75	42.1 ± 2.72
	g/L	11.7 ± 0.62	12.8 ± 0.76 ^b^	11.7 ± 1.23	12.3 ± 1.10
α_1_-	%	4.6 ± 0.65	4.7 ± 0.67	4.2 ± 0.62	4.0 ± 0.52
	g/L	1.2 ± 0.28	1.4 ± 0.17	1.1 ± 0.22	1.2 ± 0.14
α_2_-	%	29.6 ± 0.90	27.9 ± 1.44 ^a^	29.5 ± 1.64	27.6 ± 1.11 ^a^
	g/L	7.8 ± 0.73	8.3 ± 0.41	7.9 ± 0.53	8.0 ± 0.70
β-	%	6.6 ± 0.50	6.6 ± 0.67	6.4 ± 1.14	7.3 ± 0.77
	g/L	1.8 ± 0.11	2.0 ± 0.22	1.7 ± 0.30	2.1 ± 0.12 ^b^
γ-	%	13.8 ± 0.98	16.2 ± 3.05	14.7 ± 1.26	17.2 ± 2.00 ^b^
	g/L	3.7 ± 0.55	4.8 ± 1.12 ^a^	3.9 ± 0.41	5.0 ± 0.69 ^b^
A/G		1.00 ± 0.08	0.94 ± 0.15	1.00 ± 0.10	0.94 ± 0.12

Legend: a, b—superscripts in rows and groups of animals mean statistically significant differences between the sample collections (day 0 and day 11)—^a^ *p* < 0.05, ^b^ -*p* < 0.01.

**Table 4:** The effect of peroral application of *E. faecium* AL41 on mucus quantity production in different segments of small intestine of chickens.

Mucus Production (g ± SD)	5th Day of Experiment	8th Day of Experiment	11th Day of Experiment
Duodenum			
EF	3.66 ± 1.22	3.21 ± 1.21 ^d^	4.44 ± 0.27 ^a,d^
C	2.37 ± 0.69	1.41 ± 0.26	1.72 ± 0.02 ^a^
Jejunum			
EF	3.27 ± 1.65	2.99 ± 0.44 ^e^	3.91 ± 0.25 ^b,e^
C	2.82 ± 3.38	1.82 ± 0.17	1.66 ± 0.07 ^b^
Ileum			
EF	3.70 ± 1.71	3.77 ± 1.37	4.11 ± 0.44 ^c^
C	1.12 ± 4.51	1.78 ± 0.18	1.33 ± 0.60 ^c^

Legend: The mucus quantity production in g ± SD (gram ± standard deviation); EF—experimental group with *E. faecium* AL41 (CCM 8558) application; C—control group; the same letters mean the significant difference in order: ^a,b,c^ at the level *p* < 0.001; ^d^ at the level *p* < 0.05; ^e^ at the level *p* < 0.01.

**Table 5:** The numbers of *E. faecium* AL41 = CCM8558, total enterococci and coliforms in faeces (log_10_ cfu/g) (*n =* 10). Means with different superscripts are significantly different * *p* < 0.05.

Faeces	Control	EF
0 day		
EFAL41	nt	nt
Enterococci	6.46 (0.48)	6.43 (0.47)
Coliform	7.1 (0.0)	6.92 (0.84)
8 day		
EFAL41	nt	2.60 (1.0)
Enterococci	6.54 (0.81)	5.29 (0.23) *
Coliform	6.94 (0.74)	7.1 (0.0)
11 day		
EFAL41	nt	2.17 (0.31)
Enterococci	6.48 (0.81)	5.09 (0.70) *
Coliform	6.91 (0.84)	6.98 (0.83)

**Table 6:** The numbers of *E. faecium* AL41 = CCM8558, total enterococci and coliforms in cecum (log_10_ cfu/mL) (*n =* 10). Means with different superscripts are significantly different * *p* < 0.05.

Cecum	Control	EF
8 day		
EFAL41	nt	2.76 (0.43)
Enterococci	6.40 (0.81)	5.63 (0.75) *
Coliform	6.79 (0.83)	6.81 (0.83)
11 day		
EFAL41		2.16 (0.33)
Enterococci	4.61 (2.01)	4.46 (1.37)
Coliform	5.69 (0.75)	5.87 (1.42)
